# Supplementary material for: Isolable zero-valent Ditin(0) and Diplumbum(0) complexes
Source: Nat Commun. 2025 Mar 16;16:2588. doi: 10.1038/s41467-025-57568-1 (PMC11911417; doi:10.1038/s41467-025-57568-1)
Supplement: Supplementary file 1 — Supplementary Information [file 41467_2025_57568_MOESM1_ESM.pdf]

Supporting Information

for

Isolable Zero-valent Ditin(0) and Diplumbum(0) Complexes

Jinghang Shen,[1] Zhengting Zhang,[1] Xiaokang Ke,[1] Luming Peng,[1] Qianyi Zhao,[2]

Congqing Zhu,[1]* and Qin Zhu[1]*

Affiliations:

[1] State Key Laboratory of Coordination Chemistry, Jiangsu Key Laboratory of Advanced Organic Materials, School of Chemistry and Chemical Engineering, Nanjing University, Nanjing 210023, China.

[2] School of Chemistry and Chemical Engineering, Henan Normal University, Xinxiang, Henan 453007, China.

* Correspondence and requests for materials should be addressed to Q.Z. (E-mail: zhuqin@nju.edu.cn) or C.Z. (E-mail: zcq@nju.edu.cn).

Contents

1. **Supplementary Notes…………………………………………..………..S2**
2. **Supplementary Figures…………………………………….……………...S4**
3. **Supplementary Tables……………………………………………………S21**
4. **Supplementary References………………………………………………...S27**

1. Supplementary Notes

Supplementary Note 1: All manipulations were performed under an argon atmosphere using standard Schlenk techniques or in a glovebox. Commercially available chemicals were used as received without further purification. The solvents were obtained by passing through a Solve Purer G5 (MIKROUNA) solvent purification system and further dried over 4 Å molecular sieves. Deuterated solvents were dried over Na/K (C_6D_6, THF-d_8) and stored under an argon atmosphere prior to use. Nuclear magnetic resonance spectroscopy was performed using a Bruker AVIII-400 (^{1}H 400 MHz; ^{13}C{^{1}H} 101 MHz; ^{31}P{^{1}H} 162 MHz) at room temperature. The ^{1}H and ^{13}C{^{1}H} NMR chemical shifts (δ) are relative to tetramethylsilane, and ^{31}P{^{1}H} NMR chemical shifts are relative to 85% H_3PO_4. Absolute values of the coupling constants are provided in Hertz (Hz). Multiplicities are abbreviated as singlet (s), doublet (d), triplet (t), multiplet (m), and quartet (q). Solid-state ^{119}Sn NMR spectra were recorded under conditions of magic angle spinning (MAS) at 9.4 T using a Bruker Avance III NMR spectrometer equipped with a 4.0 mm double tuned MAS probe. An excitation pulse of 1.7 μs, corresponding to a flip angle of π/2, and a recycle delay of 10 s were used in the ^{119}Sn single pulse MAS NMR experiments. For ^{1}H→^{119}Sn cross polarization (CP) MAS NMR experiment, a contact time of 3 ms and a recycle delay of 2 s were applied. ^{1}H decoupling (rf power: 66 kHz) was used in the data acquisition for all experiments. Elemental analyses (C, H, N) were performed on Vario MICRO cube elemental analyzer at the Center of Modern Analysis Nanjing University. UV-vis absorption spectra were collected at 25 °C with a UV3600. Spectra were background corrected against blank THF sample measured under identical conditions. Trilithium salt $N\{CH_2CH_2NLiP^iPr_2\}_3$ was prepared according to reported procedures.[1]

Supplementary Note 2: Single-crystal X-ray diffraction data for complexes **1**, **2**, **3**, **4,** and **5** were collected on BRUKER D8 VENTURE PHOTON II detector with a radiation source of Mo(Kα) (0.71073 Å) or Ga(Kα) (1.34139 Å). All structures were solved by Patterson methods and refined on F^2 using full-matrix least-squares methods with SHELXTL-2014 program package.[2-3] All non-hydrogen atoms were refined on F^2 by full-matrix least-squares procedures with the use of anisotropic displacement parameters. Hydrogen atoms were introduced at their geometric positions and refined as riding atoms. There is a B-level alarm for structure **4** because of low bond precision on C-C bonds, which due to the twin structure and badly disorder on iPr and THF moieties when

evaluating the crystallographic information file (CIF) of complex **4** using the CheckCIF routine at www.checkcif.iucr.org. CCDC-2330637 (**1**), 2330638 (**2**), 2330636 (**3**), 2330639 (**4**), and 2377475 (**5**) contain the crystallographic data for this paper. These data can be obtained free of charge from The Cambridge Crystallographic Data Centre via www.ccdc.cam.ac.uk/data-request/cif. Details regarding the data collection and refinement for these complexes were given in Tables S1-S2.

Supplementary Note 3: Geometry optimizations were carried out by the Gaussian 16 package[4] with the PBE0 functional[5] in the gas phase. The 6-31g(d) basis set was used for C, H, B, O, and N atoms.[6-7] LanL2DZ was employed to describe Cl, P, Sn, and Pb atoms.[8] Polarization functions were added for P (ζ(d) = 0.340), Cl (ζ(d) = 0.514), Sn (ζ(f) = 0.183), Pb (ζ(f) = 0.164).[9] Supplementary Table 8 suggest that the PBE0-D3BJ[10]/6-31g(d)~LanL2DZ method provides well-optimized structural parameters with the small relative mean deviation (RD) in comparison with other DFT functionals. Frequency calculations were performed to identify the local minimum geometry of optimized structure (zero imaginary frequency). Single point energies were computed on the optimized geometries with the PBE0 functional and 6-311++G (d,p)[11] for C, H, and N atoms, and SDD basis set[12] for P, Cl, Sn and Pb atoms. The principal interacting orbital (PIO) analyses[13-14] were calculated by NBO 3.0 program[15] at the level of PBE0-D3BJ/6-311++g(d,p)~SDD based on the optimized structure. Time dependent density functional theory (TD-DFT) were performed to calculate UV-Vis spectra, under TDB3LYP-D3BJ/6-311++g(d,p)~SDD level[16] including solvent effect in a polarizable continuum model (PCM, solvent = THF)[17]. The electrostatic potential analysis, ADCH charge analysis, atoms in molecules analysis, noncovalent interactions (NCI) analysis, and natural adaptive orbital (NAdO) were carried out via the Multiwfn[18] and visualized by VMD programs.[19]

2. Supplementary Figures

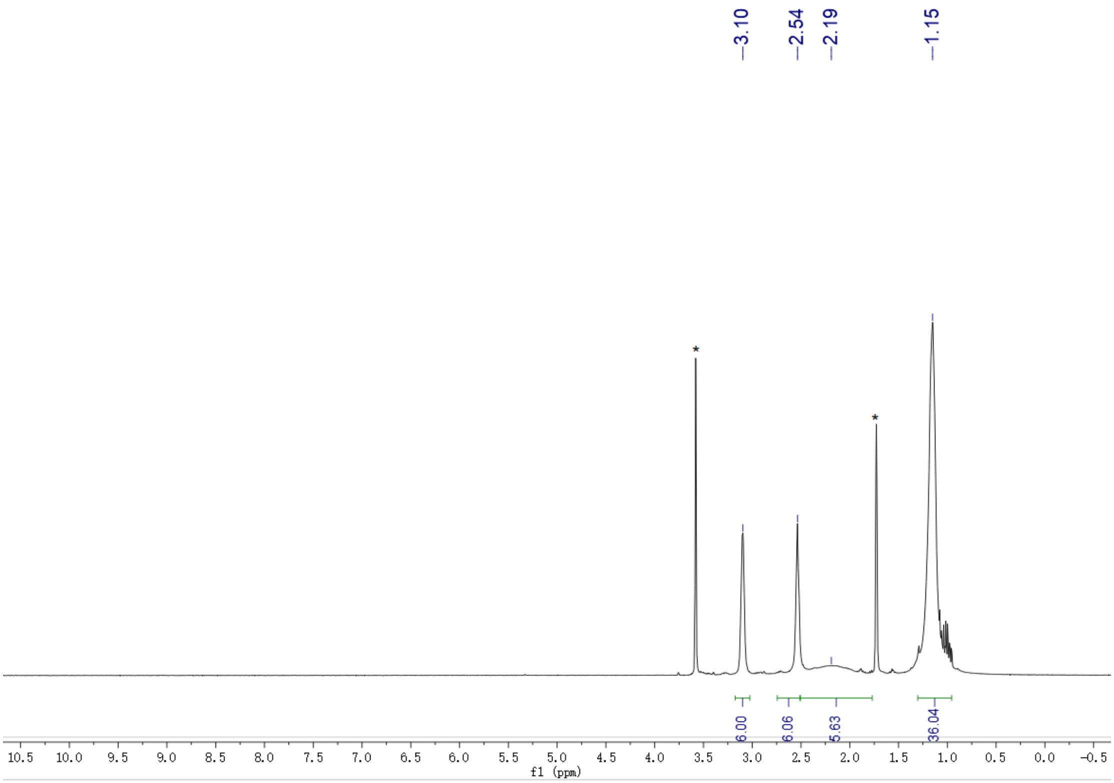

Supplementary Figure 1. The ^{1}H NMR (400 MHz, THF-d_8) spectrum of **1**. (*: residue of THF)

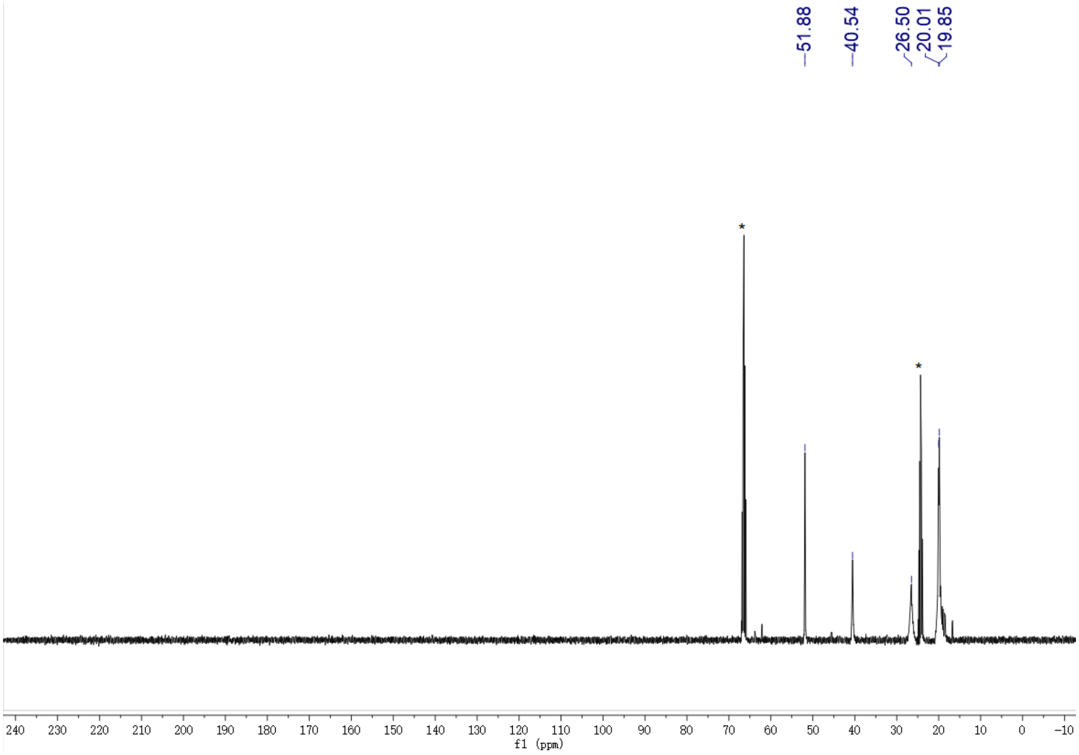

Supplementary Figure 2. The $^{13}C\{^{1}H\}$ NMR (101 MHz, THF-d_8) spectrum of **1**. (*: residue of THF)

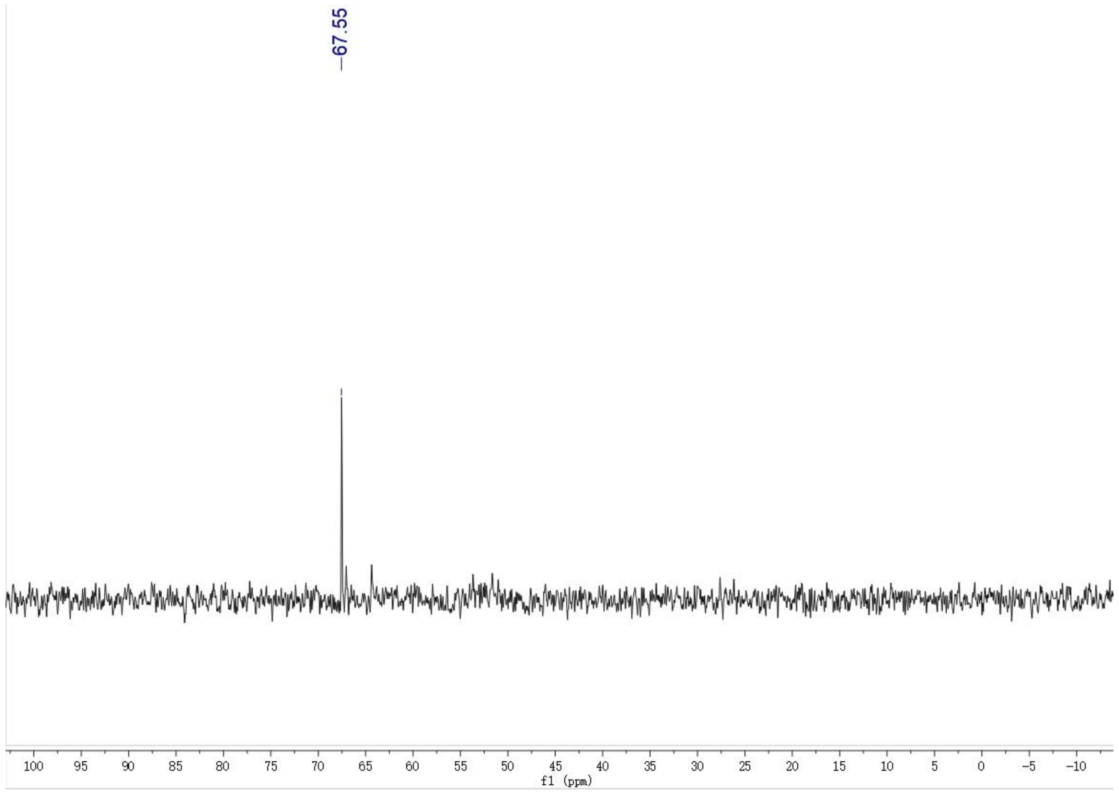

Supplementary Figure 3. The $^{31}P\{^1H\}$ NMR (162 MHz, THF-d_8) spectrum of **1**.

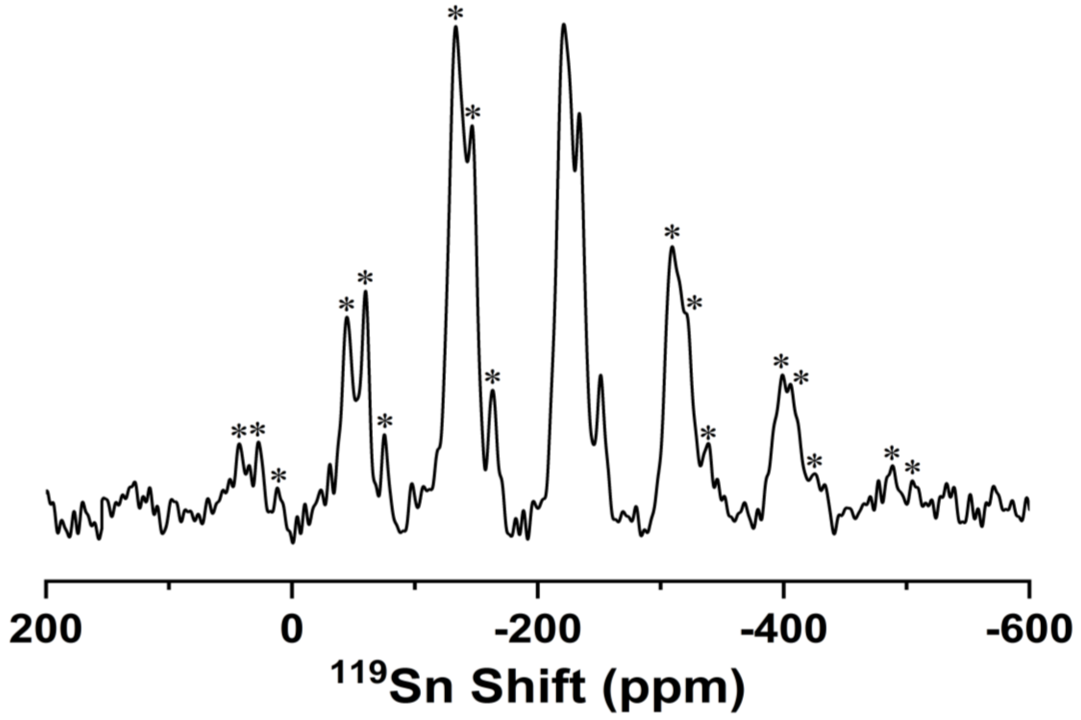

Supplementary Figure 4. The single pulse ^{119}Sn solid-state NMR spectrum of **1**. MAS rate: 13 kHz; recycle delay: 10 s. * denotes the spinning sidebands.

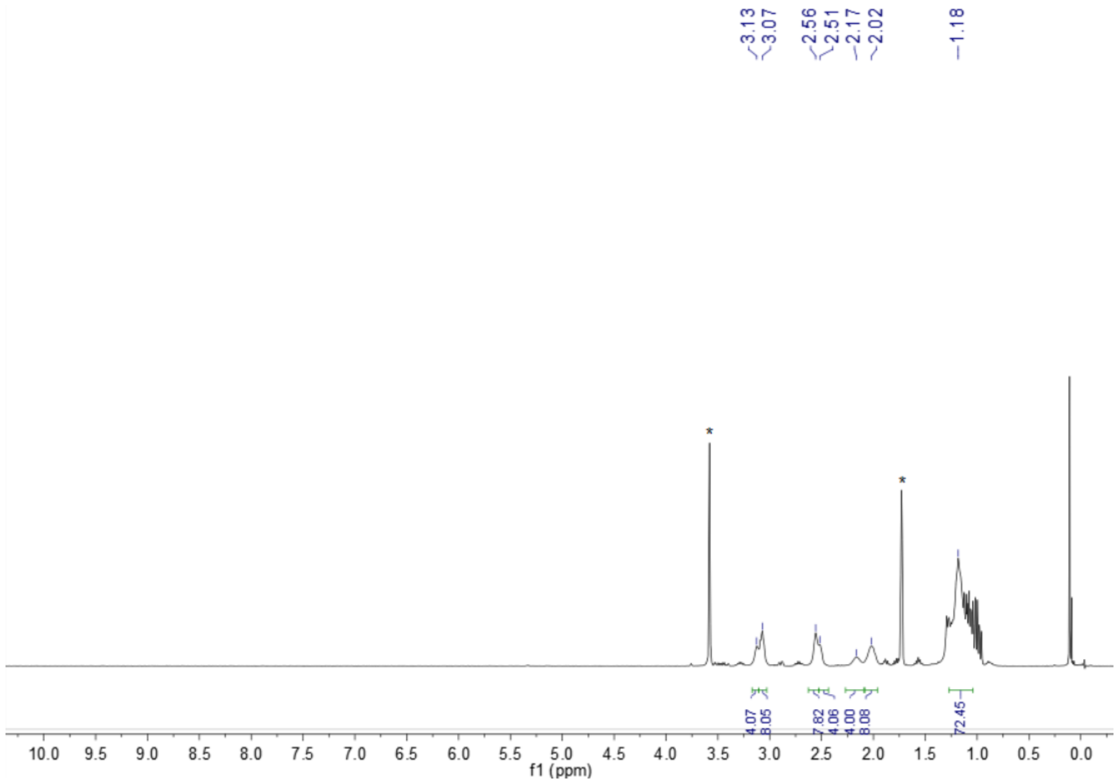

Supplementary Figure 5. The ^{1}H NMR (400 MHz, THF-d_8) spectrum of **2**. (*: residue of THF)

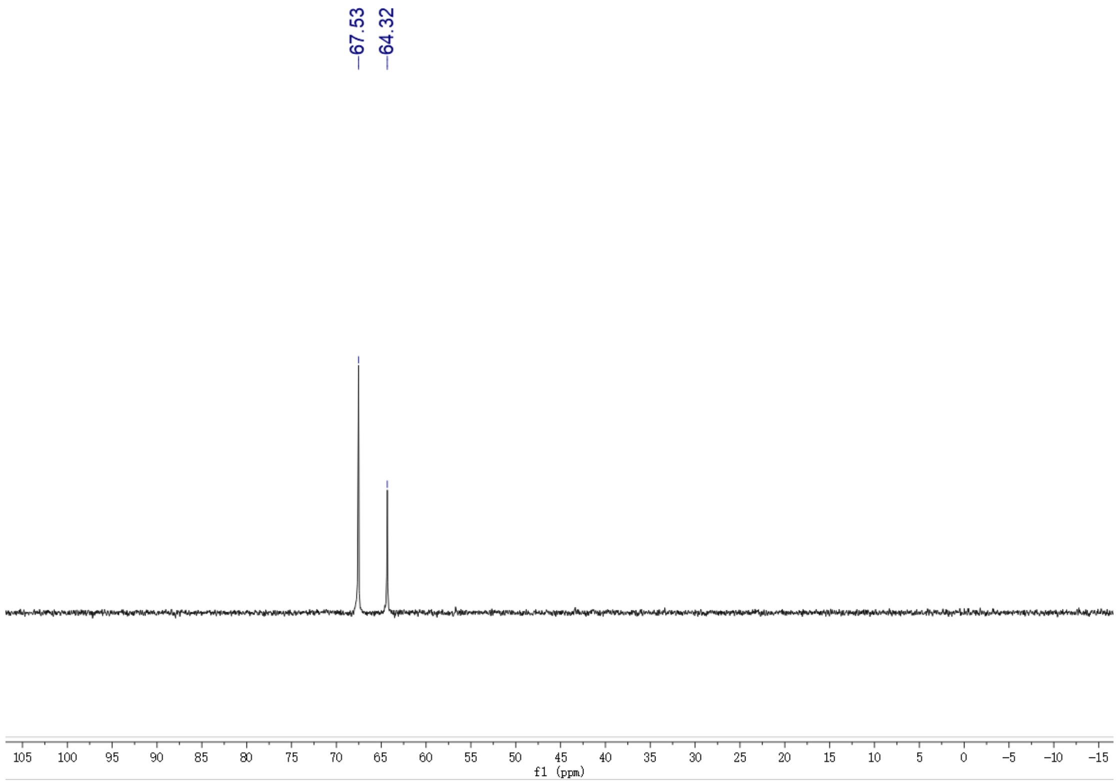

Supplementary Figure 6. The $^{31}P\{^{1}H\}$ NMR (162 MHz, THF-d_8) spectrum of **2**.

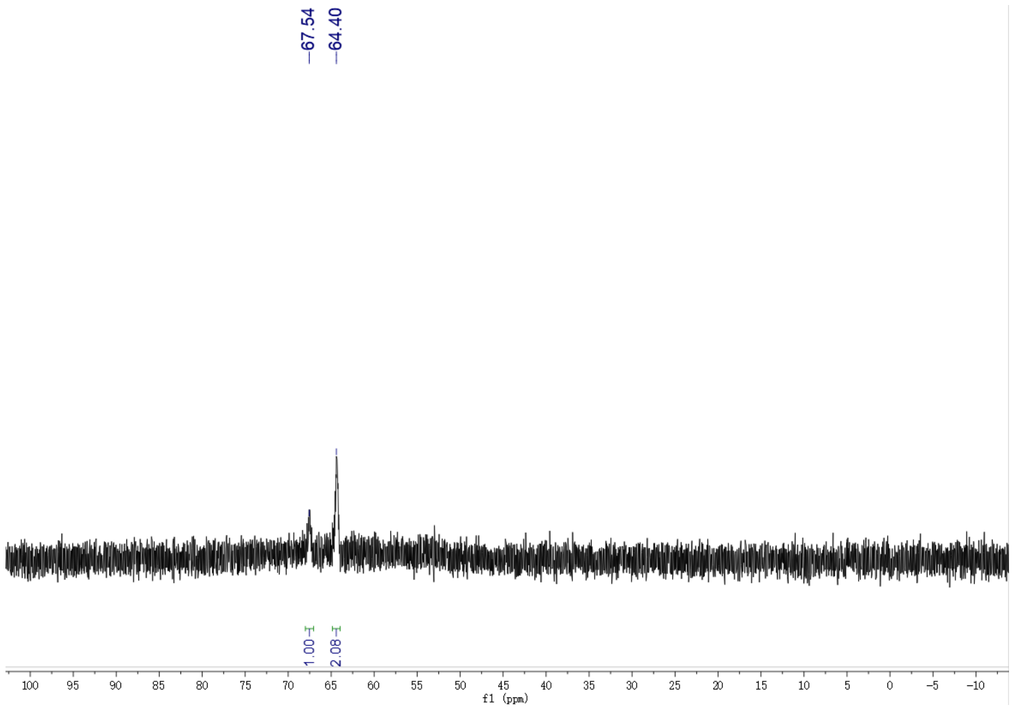

Supplementary Figure 7. The ^{31}P NMR (162 MHz, THF-d_8) spectrum of **2**.

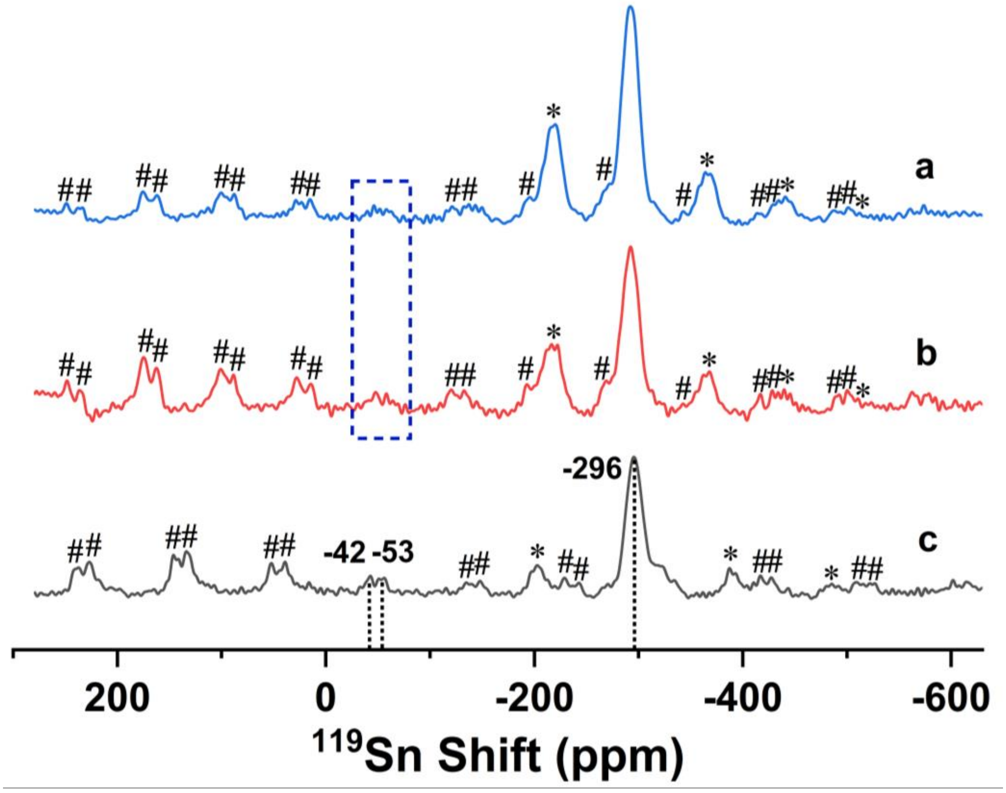

Supplementary Figure 8. The ^{119}Sn solid-state NMR spectra of **2**. (**a**) $^{1}H \rightarrow ^{119}Sn$ CP-MAS NMR spectrum. MAS rate: 11 kHz. (**b**) ^{119}Sn single-pulse MAS NMR spectrum. MAS rate: 11 kHz. (**c**) ^{119}Sn single-pulse MAS NMR spectrum. MAS rate: 14 kHz. * and # denote spinning sidebands of the resonances at -296 and -42/-53 ppm, respectively.

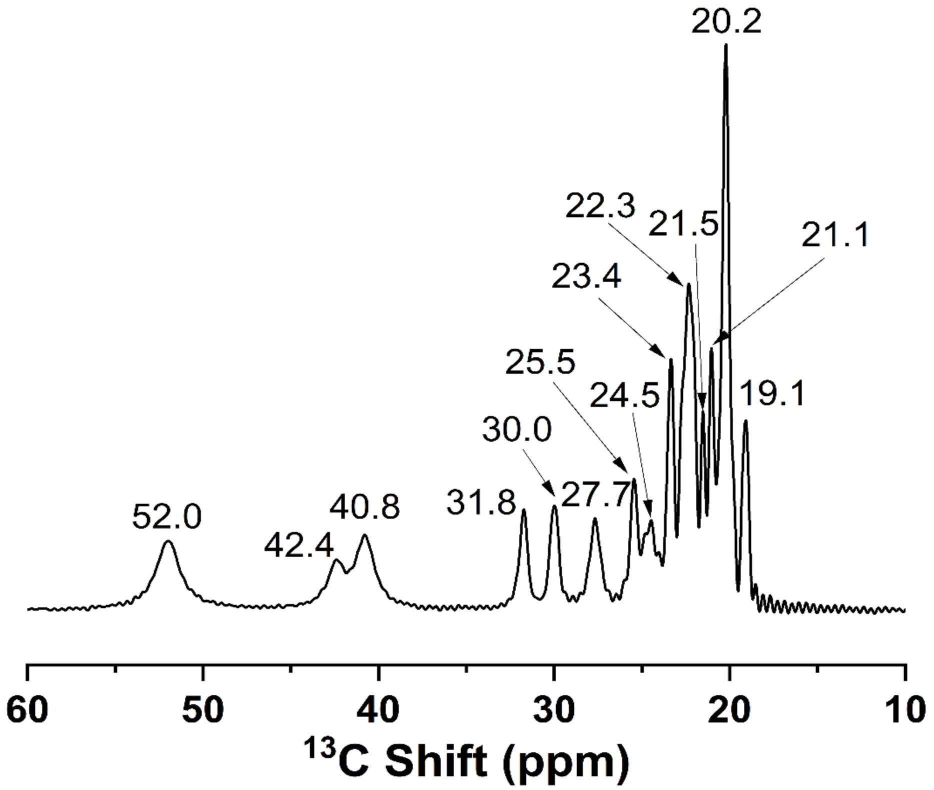

Supplementary Figure 9. The $^{1}H \rightarrow ^{13}C$ CP-MAS NMR spectrum of **2**. MAS rate: 14 kHz.

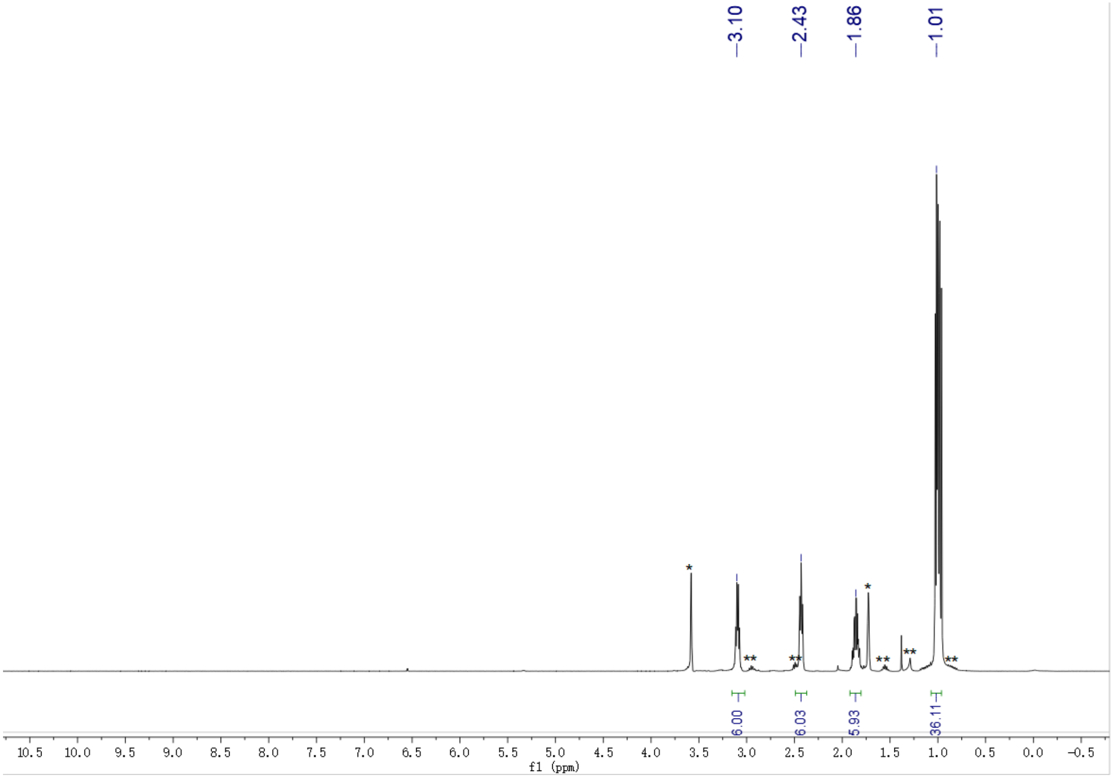

Supplementary Figure 10. The ^{1}H NMR (400 MHz, THF-d_8) spectrum of **3**. (*: residue of THF, **: protonated ligand)

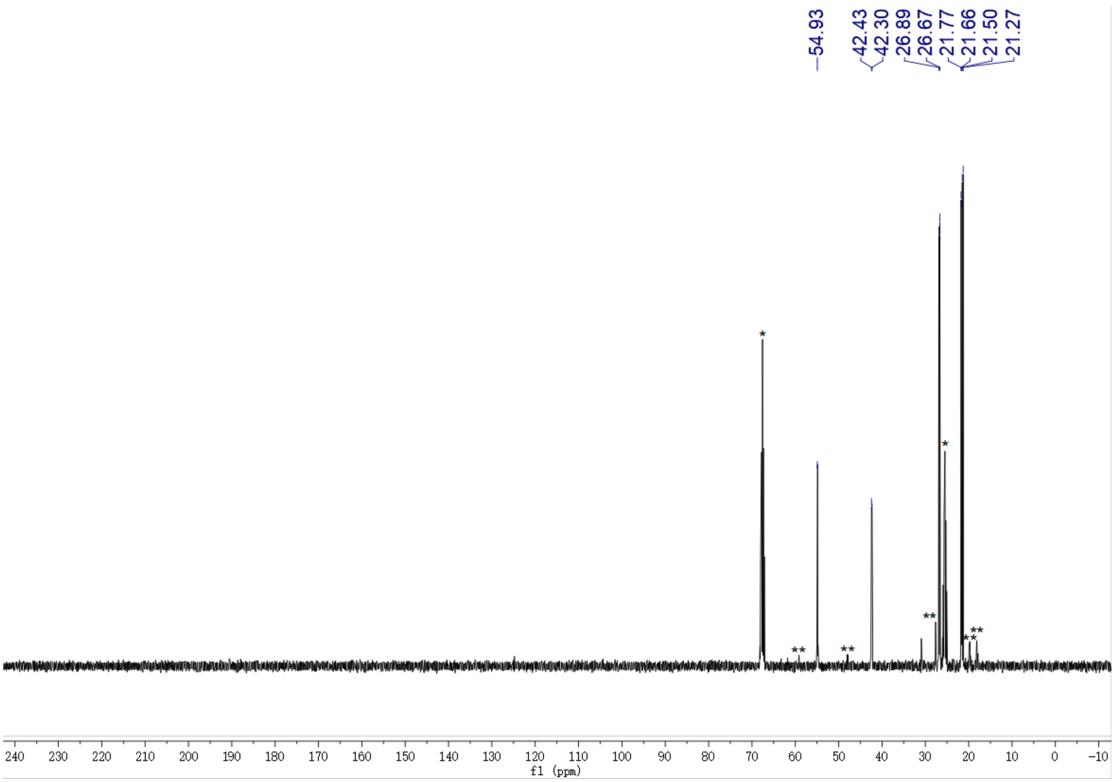

Supplementary Figure 11. The $^{13}C\{^{1}H\}$ NMR (101 MHz, THF-d_8) spectrum of **3.** (*: residue of THF, **: protonated ligand)

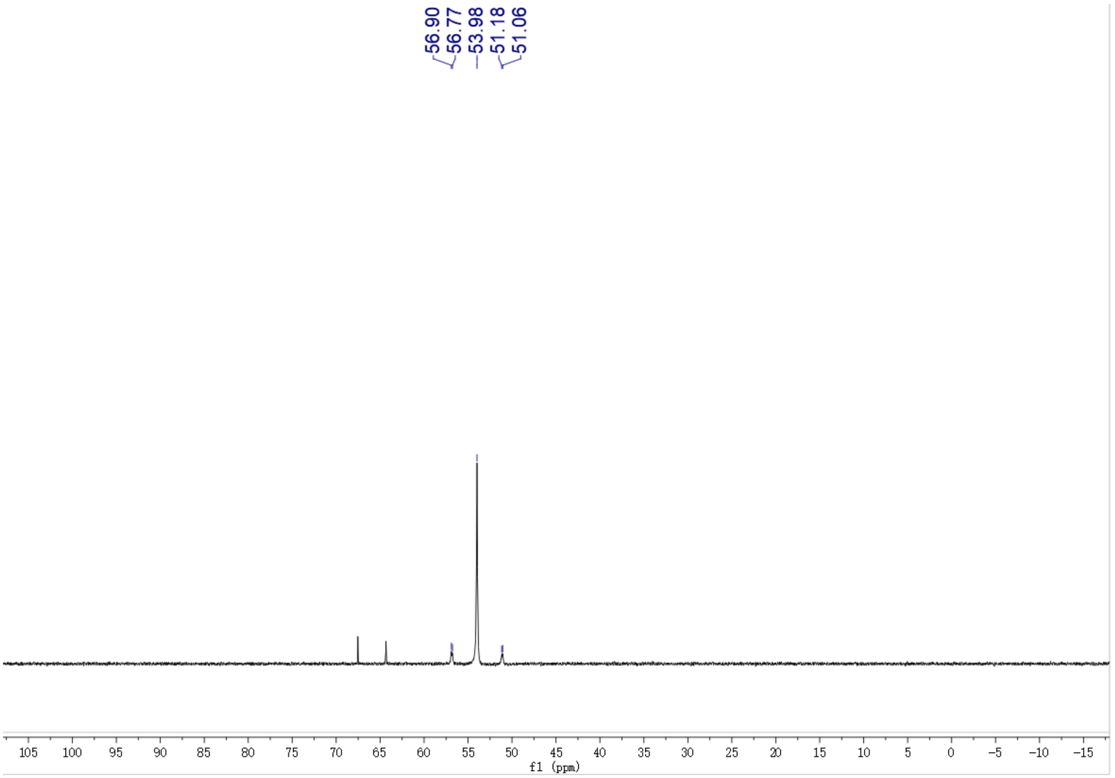

Supplementary Figure 12. The $^{31}P\{^{1}H\}$ NMR (162 MHz, THF-d_8) spectrum of **3**.

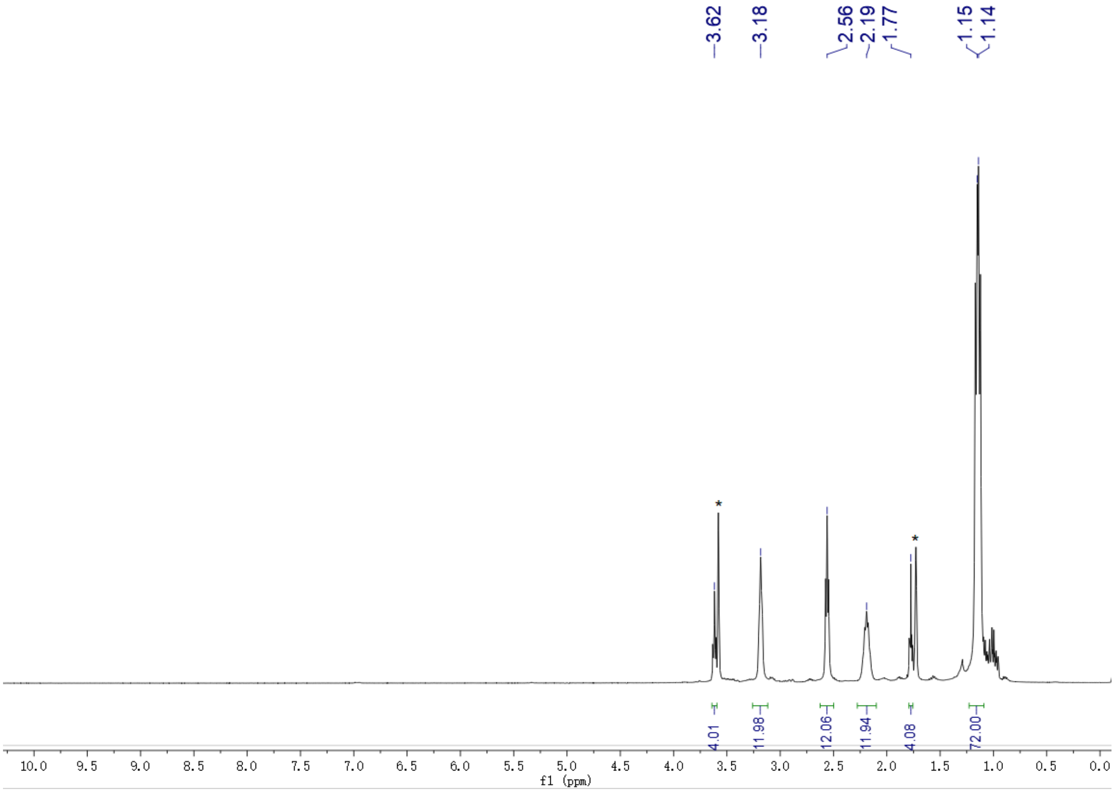

Supplementary Figure 13. The ^{1}H NMR (400 MHz, THF-d_8) spectrum of **4**. (*: residue of THF)

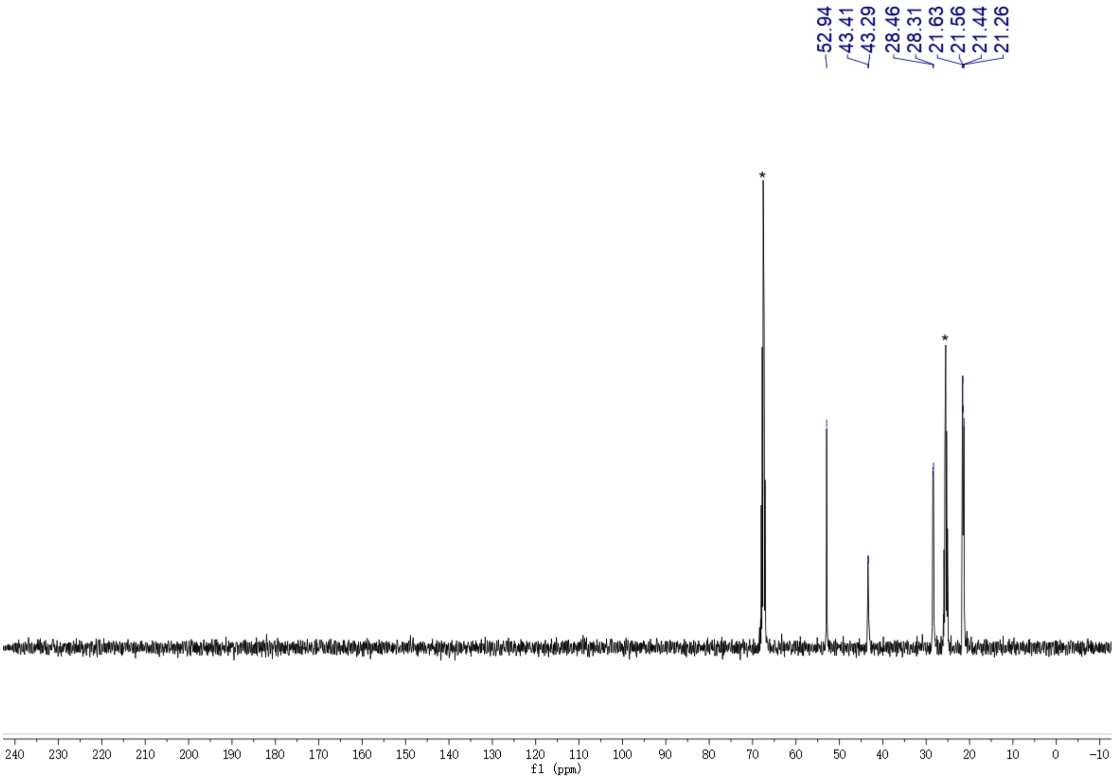

Supplementary Figure 14. The $^{13}C\{^{1}H\}$ NMR (101 MHz, THF-d_8) spectrum of **4**. (*: residue of THF)

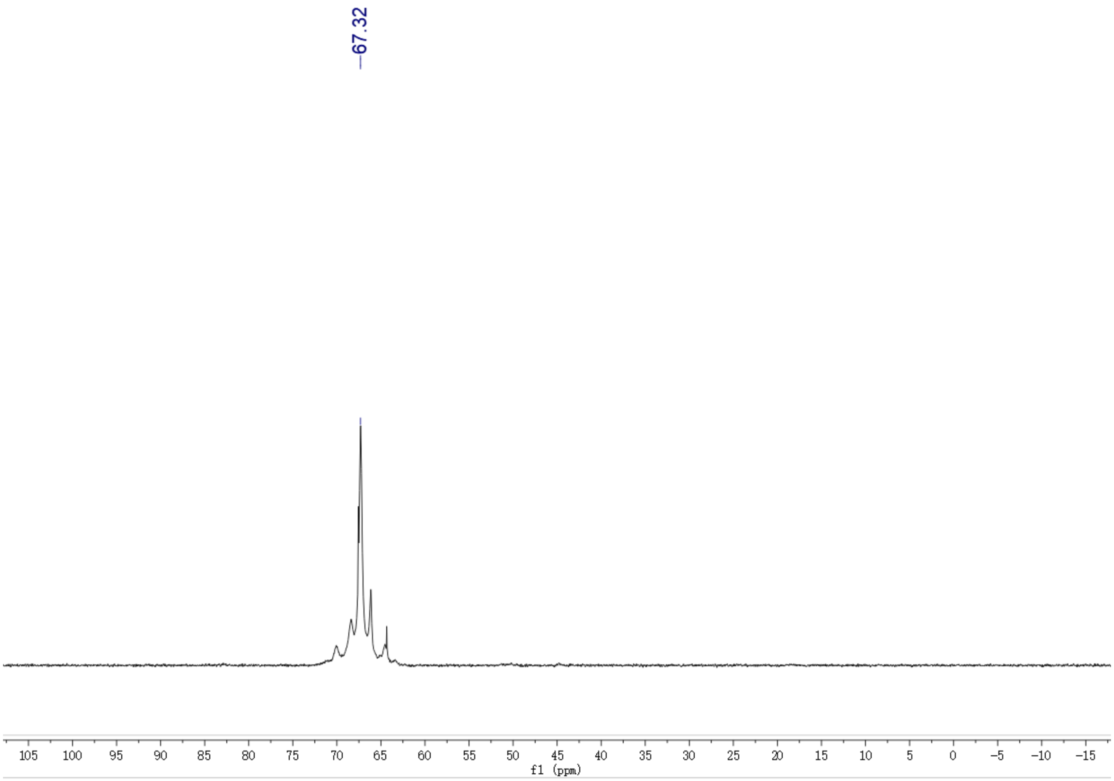

Supplementary Figure 15. The $^{31}P\{^{1}H\}$ NMR (162 MHz, THF-d_8) spectrum of **4**.

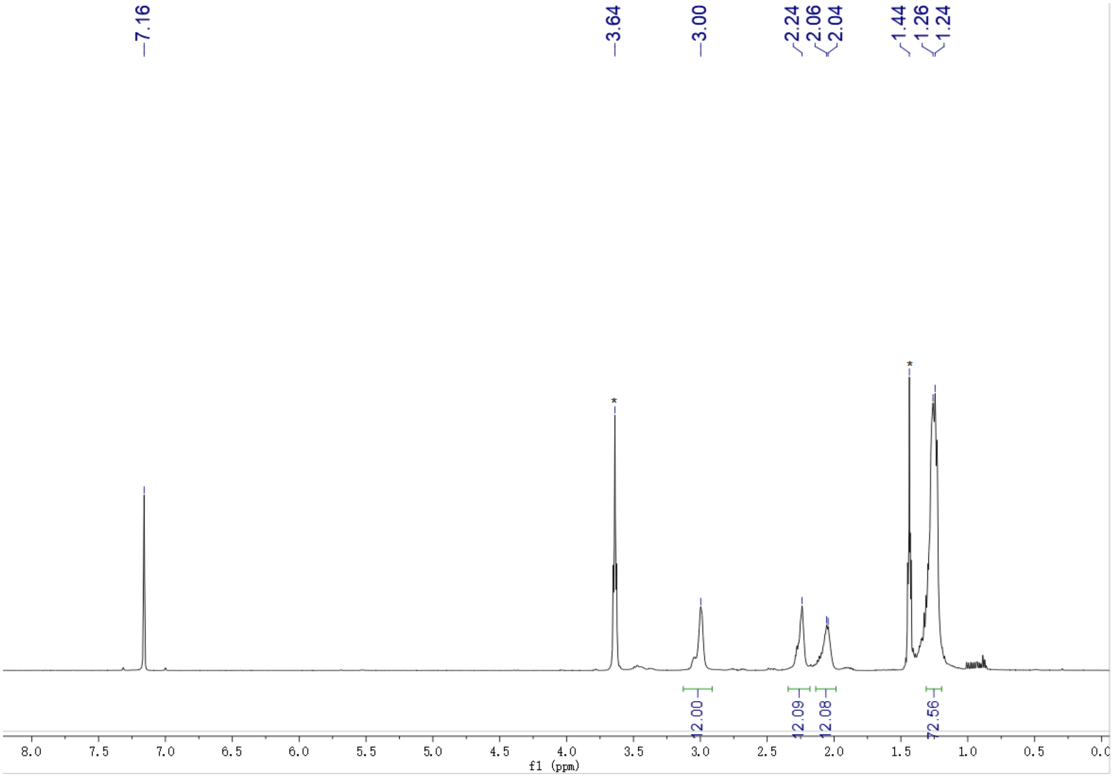

Supplementary Figure 16. The ^{1}H NMR (400 MHz, C_6D_6) spectrum of **5**. (*: residue of THF)

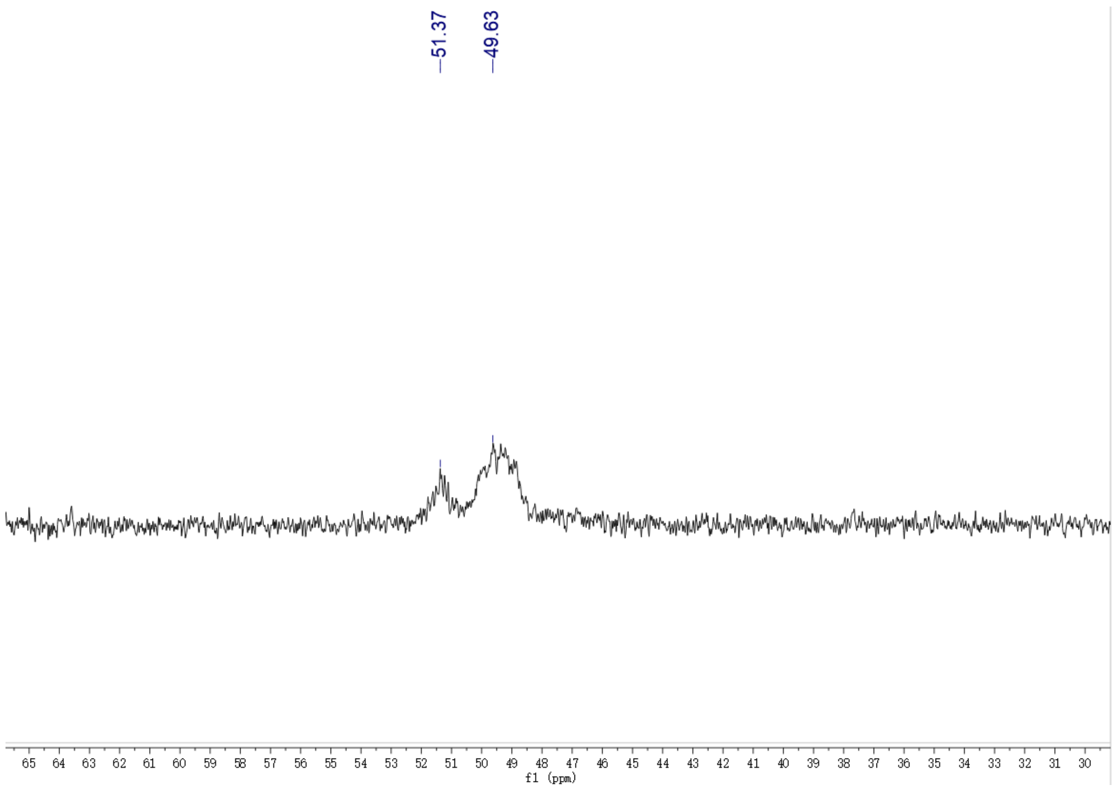

Supplementary Figure 17. The $^{31}P\{^{1}H\}$ NMR (162 MHz, THF-d_8) spectrum of **5**.

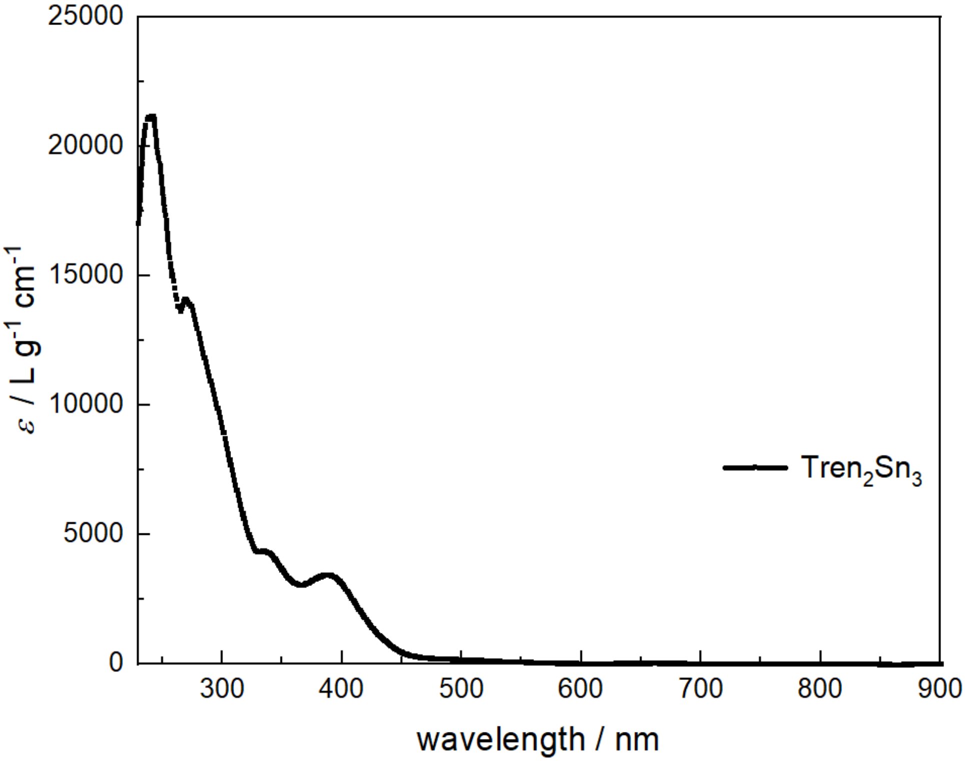

Supplementary Figure 18. UV-vis absorption spectrum of complex **1** in THF at RT.

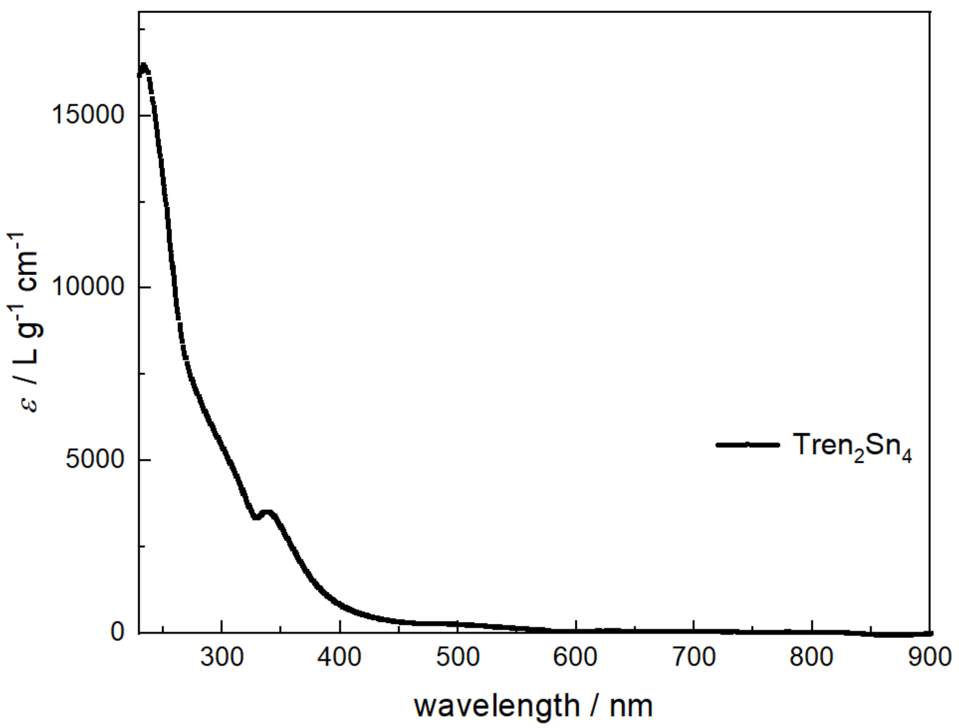

Supplementary Figure 19. UV-vis absorption spectrum of complex **2** in THF at RT.

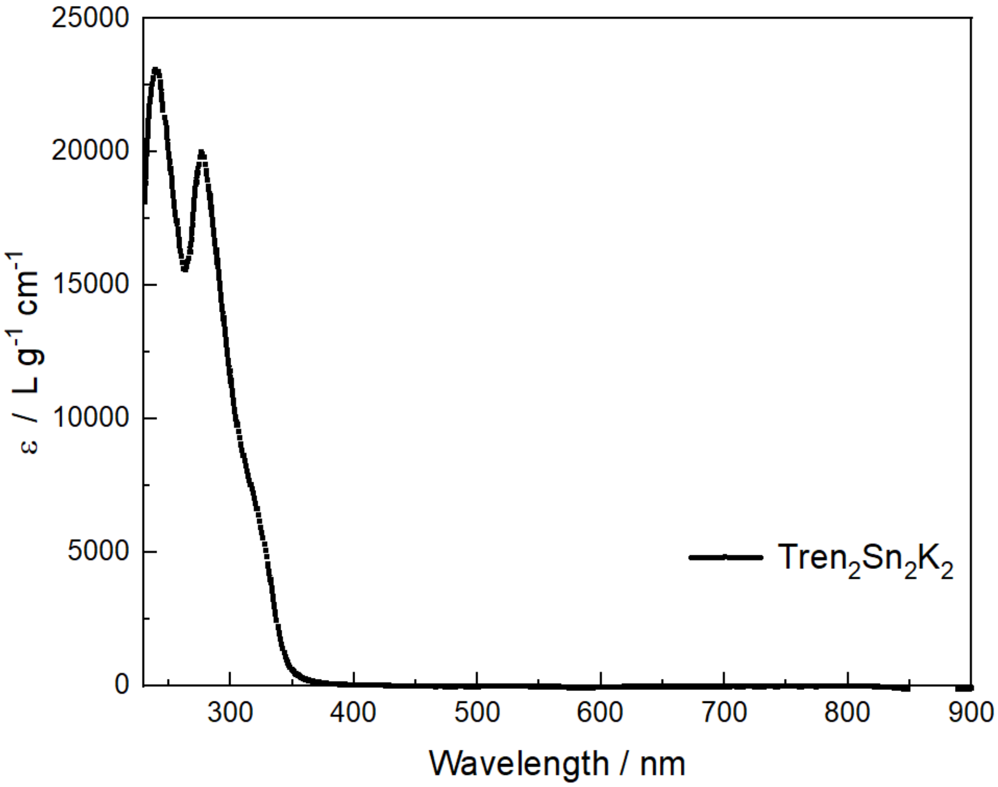

Supplementary Figure 20. UV-vis absorption spectrum of complex **3** in THF at RT.

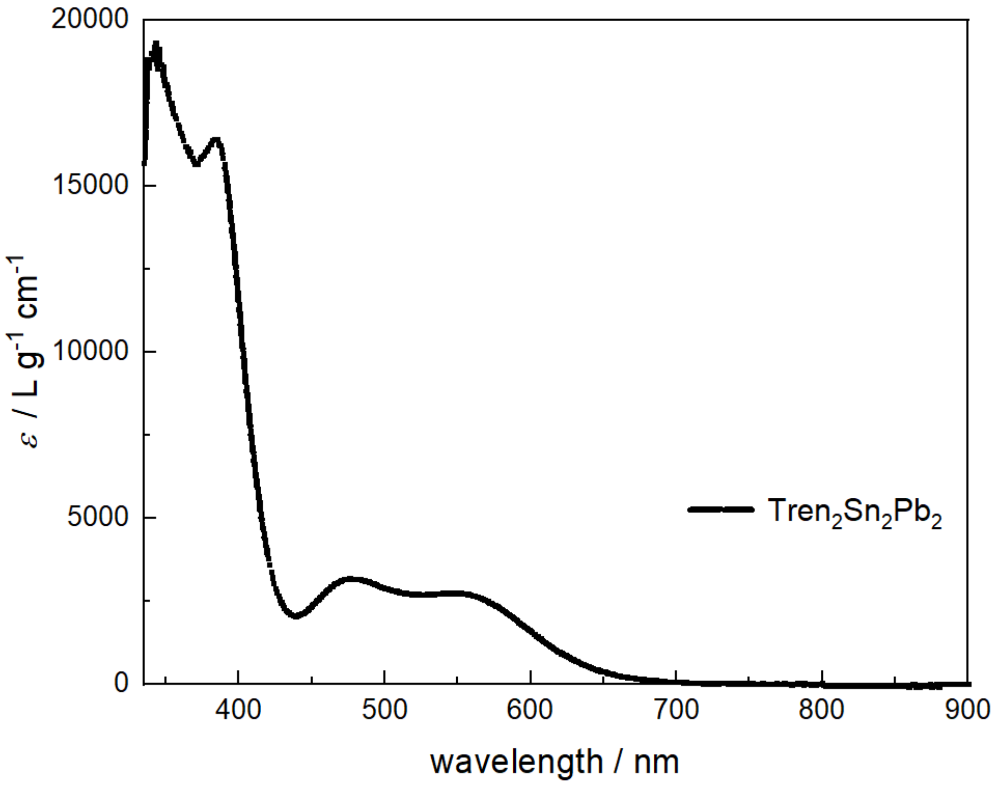

Supplementary Figure 21. UV-vis absorption spectrum of complex **4** in THF at RT.

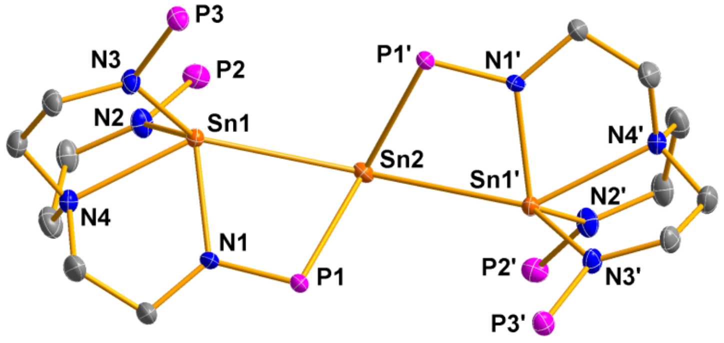

Supplementary Figure 22. Molecular structure of complex **1** (drawn with 30% probability). All the hydrogen atoms and isopropyl moieties in the $P^{i}Pr_2$ were omitted for clarity.

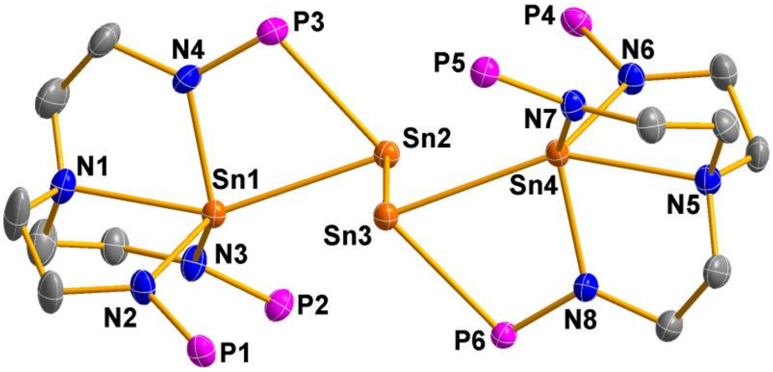

Supplementary Figure 23. Molecular structure of complex **2** (drawn with 30% probability). All the hydrogen atoms and isopropyl moieties in the P[i]Pr2 were omitted for clarity.

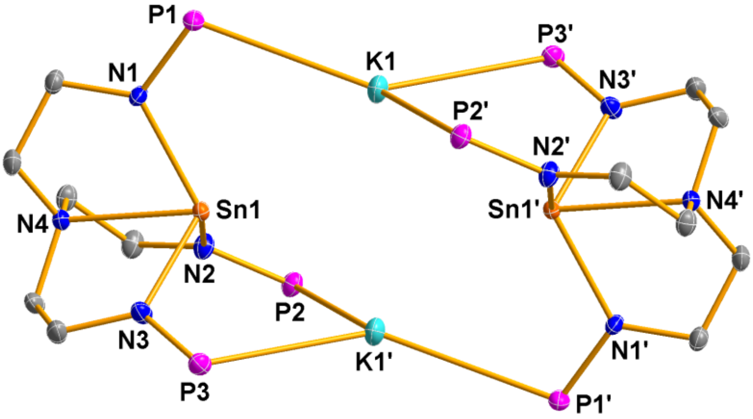

Supplementary Figure 24. Molecular structure of complex **3** (drawn with 30% probability). All the hydrogen atoms and isopropyl moieties in the P[i]Pr2 were omitted for clarity.

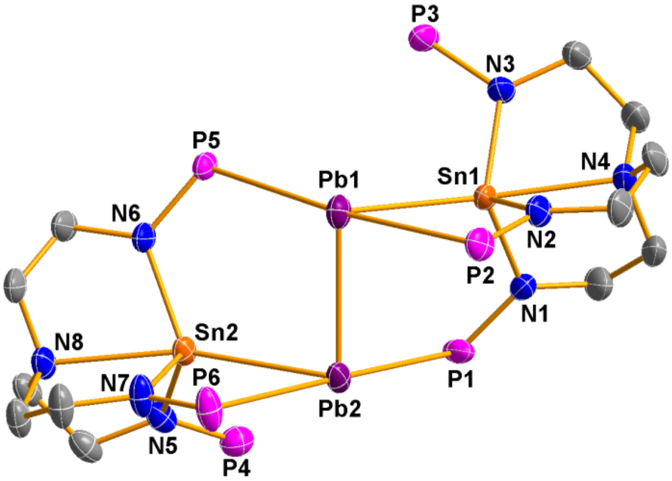

Supplementary Figure 25. Molecular structure of complex **4** (drawn with 30% probability). All the hydrogen atoms, solvents and isopropyl moieties in the P[i]Pr2 were omitted for clarity.

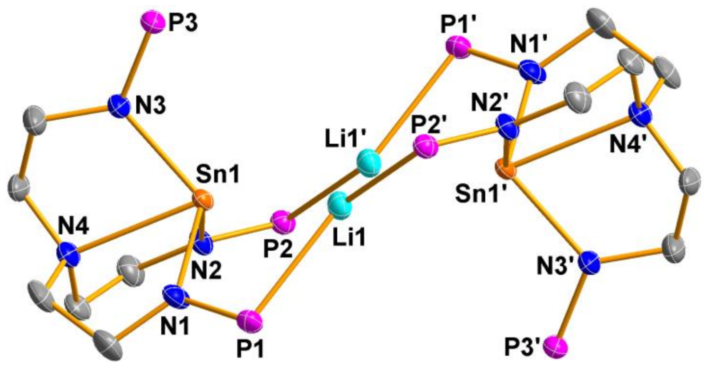

Supplementary Figure 26. Molecular structure of complex **5** (drawn with 30% probability). All the hydrogen atoms and isopropyl moieties in the P^{i}Pr2 were omitted for clarity.

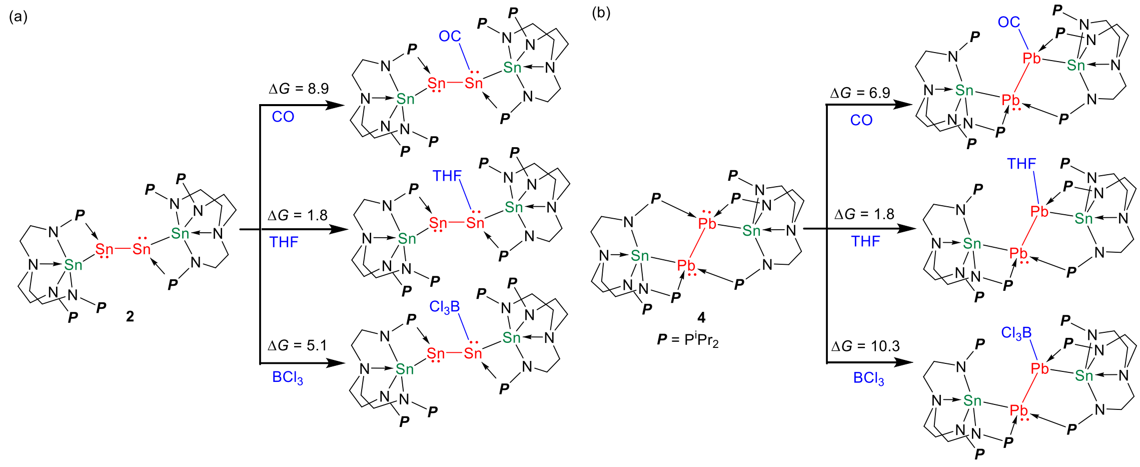

Supplementary Figure 27. Energy profile. The reaction energy profile for the interaction of complexes **2** (**a**) and (**b**) **4** with small molecules (CO, THF, and BCl_3). The free Gibbs energy change are given in kcal/mol.

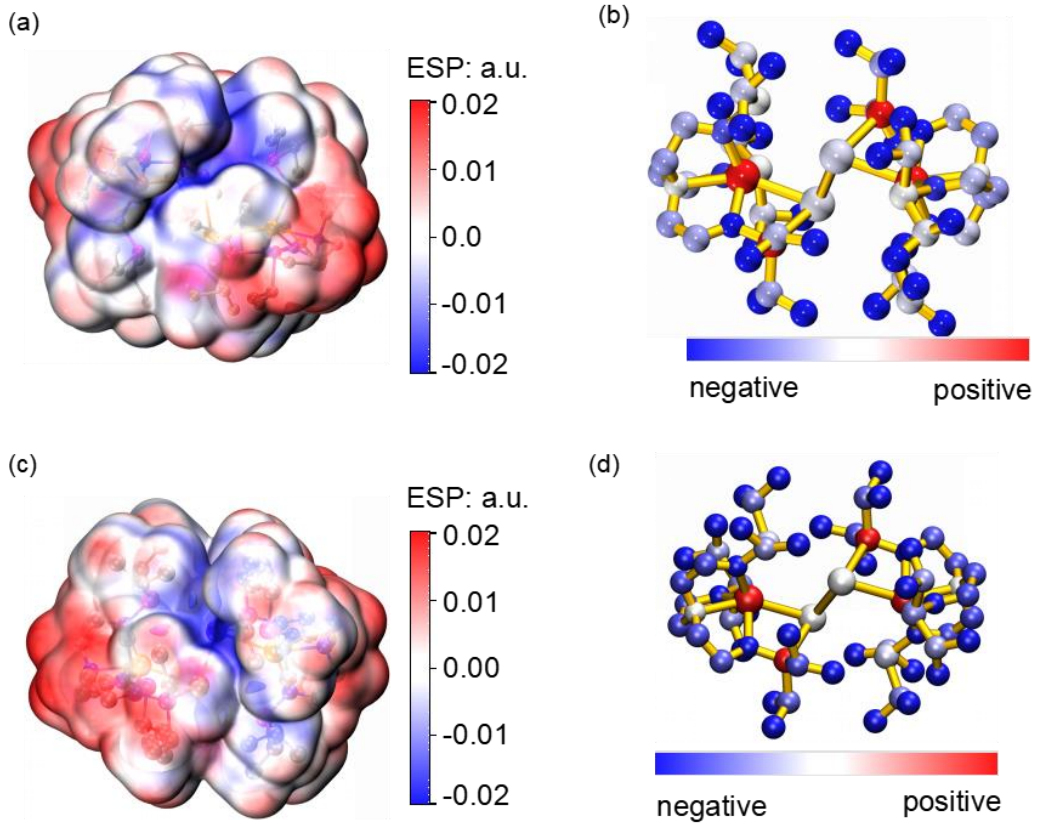

Supplementary Figure 28. Electrostatic potential and charge analyses. Electrostatic potential surface for complexes **2** (**a**) and **4** (**c**) and atomic dipole corrected Hirshfeld atomic charge analyses for complexes **2** (**b**) and **4** (**d**).

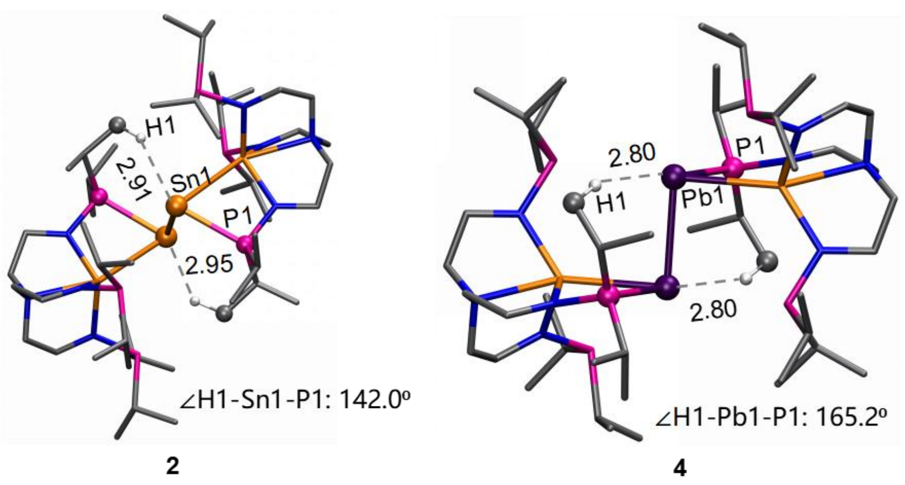

Supplementary Figure 29. The geometry of hydrogen bond distance and angle on complexes **2** and **4**. The distances were given in Å.

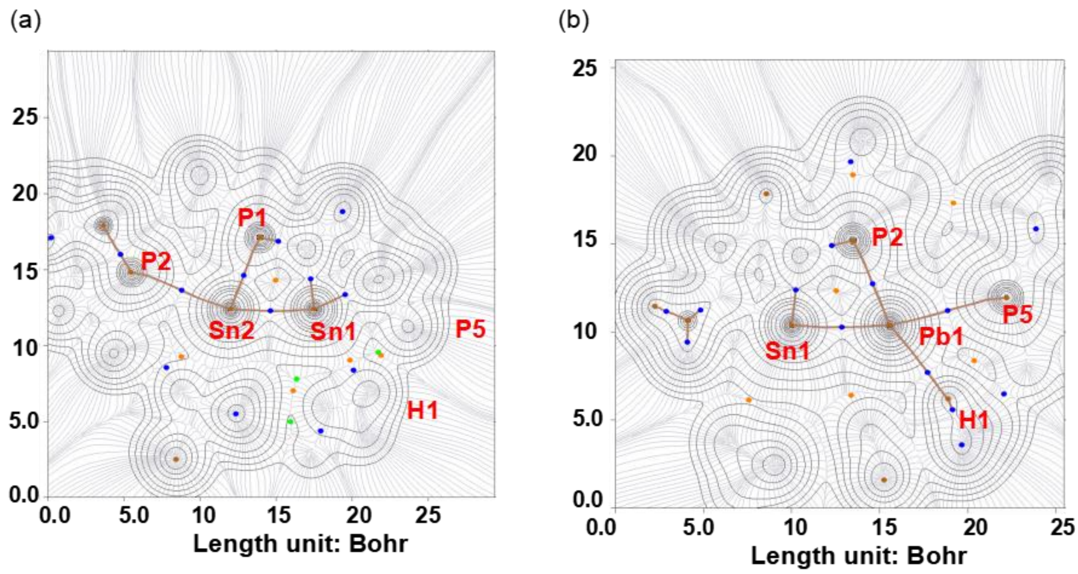

Supplementary Figure 30. Electron density map. Electron density gradient line map containing critical points and topological paths for complexes **2** (**a**) and **4** (**b**). Brown, blue, and orange dots denote (3,-3), (3,-1), and (3,+1) critical points, respectively.

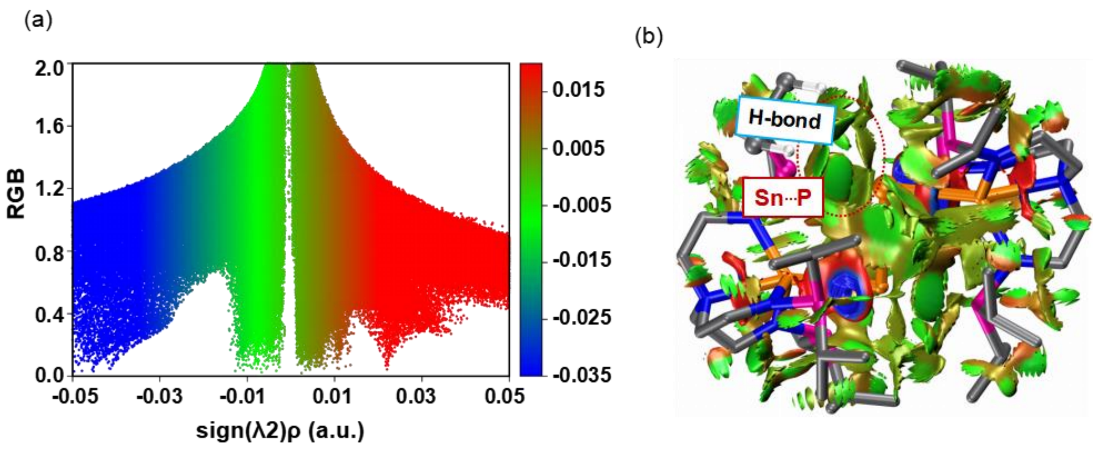

Supplementary Figure 31. Reduced density gradient and noncovalent interaction analysis. Visualization of the reduced density gradient (RDG) of complex **2** in relation to the sign(λ2)ρ (**a**) and the noncovalent interaction (NCI) analysis (**b**).

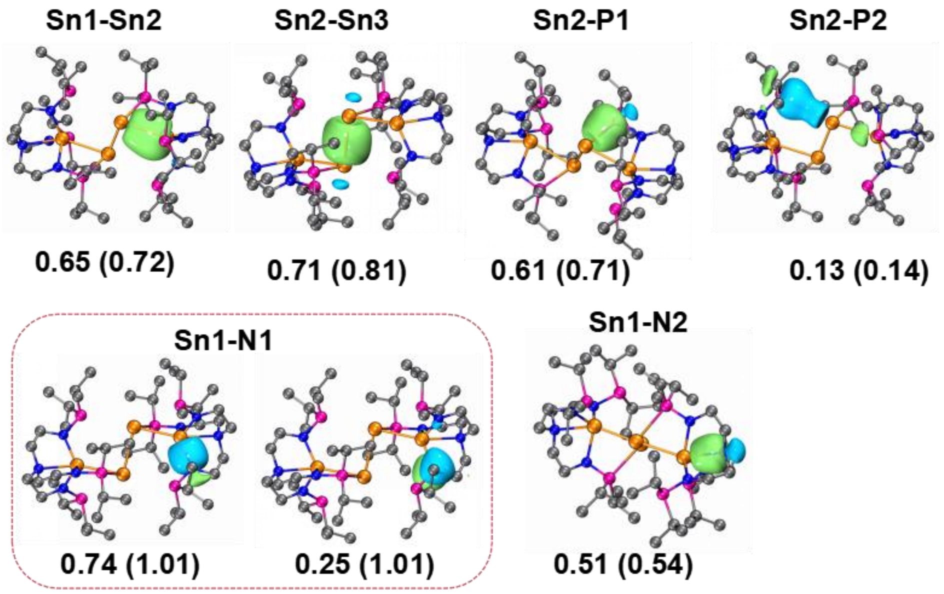

Supplementary Figure 32. Six bonding NAdOs of Sn(III)-Sn(0), Sn(0)-Sn(0), and Sn(0)-P(III) in complex **2**. Hydrogen atoms were omitted for clarity. The interaction was quantified by eigenvalue. The isosurface 0.050 a.u. was plotted.

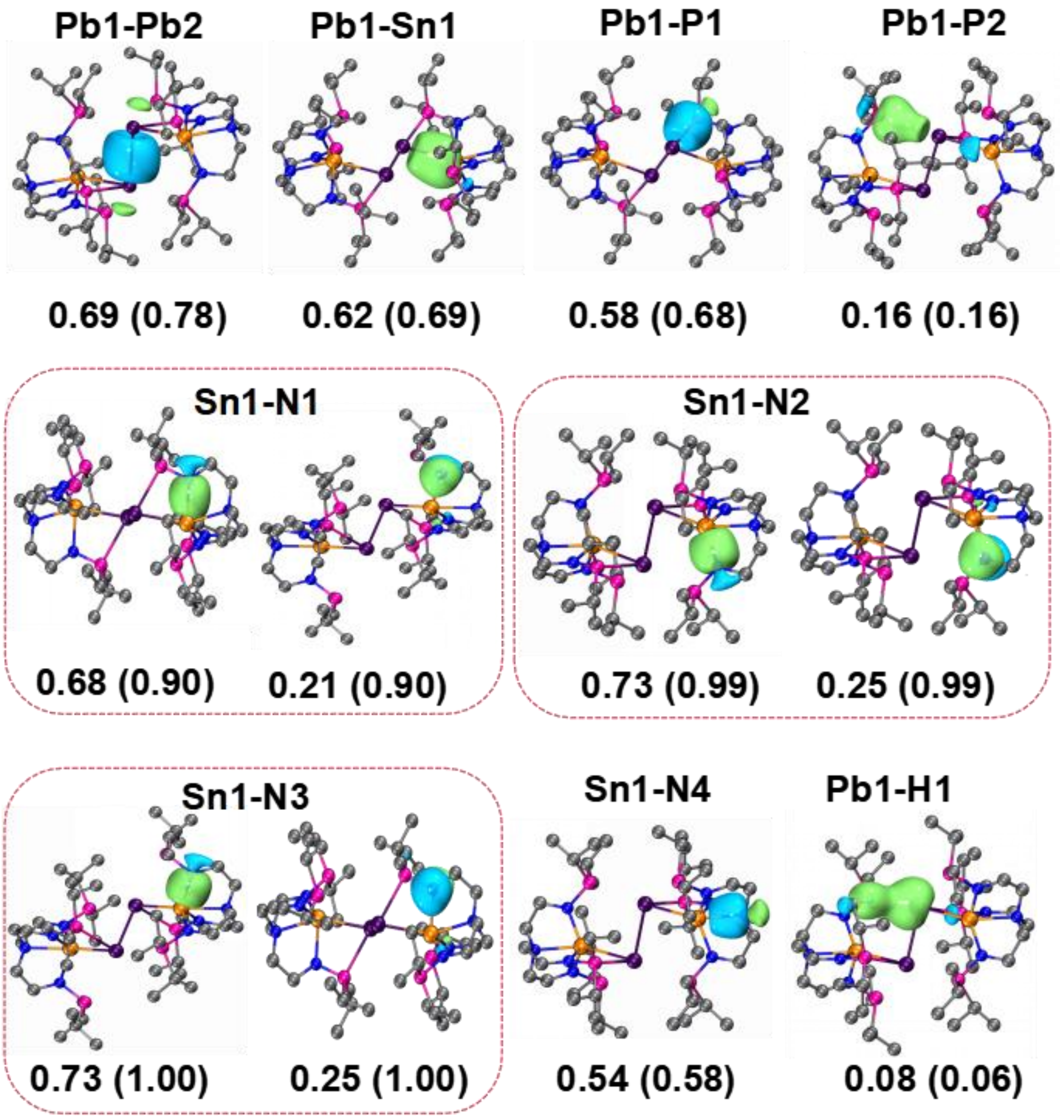

Supplementary Figure 33. Key bonding NAdOs of Pb(0)-Pb(0), Sn(III)-Pb(0), and two Pb(0)-P(III) in complex **4**. Hydrogen atoms were omitted for clarity. The interaction was quantified by eigenvalue. The isosurface 0.050 a.u. was plotted.

3. Supplementary Tables

Supplementary Table 1. Crystal data and structural refinement for **1** and **2**.

Complex	**1**	**2**
formula	$C_{48}H_{108}N_8P_6Sn_3$	$C_{48}H_{108}N_8P_6Sn_4$
Mr [g/mol]	1339.31	1458.00
Temp./K	296.15	193.0
Crystal system	Monoclinic	Monoclinic
Space group	*P*2/n	$P2_1/c$
a [Å]	11.0195(3)	23.4201(14)
b [Å]	19.9924(5)	14.8198(9)
c [Å]	29.3475(9)	20.8788(12)
α [°]	90	90
β [°]	99.8410(10)	116.133(2)
γ [°]	90	90
Volume [$Å^3$]	6370.3(3)	6505.8(7)
Z	4	4
ρ_{calc}g [cm^3]	1.396	1.489
μ [mm^{-1}]	1.354	9.217
F(000)	2768.0	2968.0
Crystal size [mm^3]	0.14 × 0.13 × 0.12	0.12 × 0.11 × 0.11
Radiation	MoKα (λ = 0.71073)	GaKα (λ = 1.34139)

2Θ range for data collection [°]	3.476 to 55.108	3.656 to 108.83
Index ranges	$-14 \leqslant h \leqslant 13, -25 \leqslant k \leqslant 26, -29 \leqslant l \leqslant 38$	$-28 \leqslant h \leqslant 28, -17 \leqslant k \leqslant 17, -25 \leqslant l \leqslant 24$
Reflections collected	54726	79949
Independent reflections	14103 [R_{int} = 0.0397, R_{sigma} = 0.0363]	12007 [R_{int} = 0.0559, R_{sigma} = 0.0342]
Data/restraints/parameters	14103/0/611	12007/0/619
Goodness-of-fit on F^2	1.037	1.049
Final R indexes [I>=2σ(I)]	R_1 = 0.0365, wR_2 = 0.1236	R_1 = 0.0426, wR_2 = 0.1074
Final R indexes [all data]	R_1 = 0.0506, wR_2 = 0.1398	R_1 = 0.0559, wR_2 = 0.1192
Largest diff. peak/hole / e Å^{-3}	0.97/-1.11	1.14/-1.62
CCDC	2330637	2330638

Supplementary Table 2. Crystal data and structural refinement for **3**, **4** and **5**.

Complex	**3**	**4**	**5**
formula	$C_{48}H_{108}K_2N_8P_6Sn_2$	$C_{52}H_{116}ON_8P_6Sn_2Pb_2$	$C_{48}H_{108}N_8P_6Sn_2Li_2$
Mr [g/mol]	1298.82	1707.10	1234.50
Temp./K	193.0	293.0	193.0

Crystal system	Monoclinic	Orthorhombic	Triclinic
Space group	$P2_1/n$	$Pna2_1$	P-1
a [Å]	12.4469(5)	26.8767(12)	11.7094(5)
b [Å]	14.4257(6)	17.3218(8)	12.7652(5)
c [Å]	18.5211(6)	14.9565(5)	13.2960(6)
α [°]	90	90	98.555(2)
β [°]	92.3360(10)	90	112.077(2)
γ [°]	90	90	113.882(2)
Volume [Å^3]	3322.8(2)	6962.9(5)	1574.22(12)
Z	2	4	1
ρ_{calc}g [cm^3]	1.298	1.628	1.302
μ [mm^{-1}]	1.057	5.707	5.361
F(000)	1360.0	3384.0	648
Crystal size [mm^3]	0.13 × 0.12 × 0.11	0.14 × 0.13 × 0.12	0.14 × 0.13 × 0.12
Radiation	MoKα (λ = 0.71073)	GaKα (λ = 0.71073)	GaKα (λ = 1.34139)
2θ range for data collection [°]	4.324 to 55.018	5.448 to 54.976	6.676 to 107.984
Index ranges	-16 ≤ h ≤ 13, -18 ≤ k ≤ 17, -23 ≤ l ≤ 24	-34 ≤ h ≤ 34, -21 ≤ k ≤ 22, -19 ≤ l ≤ 18	-14 ≤ h ≤ 13, -15 ≤ k ≤ 15, -16 ≤ l ≤ 15
Reflections collected	30100	148123	18971

Independent reflections	7617 [R_{int} = 0.0471, R_{sigma} = 0.0412]	15694 [R_{int} = 0.0711, R_{sigma} = 0.0399]	5541 [R_{int} = 0.0426, R_{sigma} = 0.0400]
Data/restraints/parameters	7617/0/310	15694/1169/982	5541/0/310
Goodness-of-fit on F^2	1.053	1.044	1.165
Final R indexes [I>=2σ(I)]	R_1 = 0.0403, wR_2 = 0.1329	R_1 = 0.0353, wR_2 = 0.0842	R_1 = 0.0431, wR_2 = 0.1276
Final R indexes [all data]	R_1 = 0.0481, wR_2 = 0.1412	R_1 = 0.0427, wR_2 = 0.0869	R_1 = 0.0478, wR_2 = 0.1324
Largest diff. peak/hole / e Å^{-3}	1.23/-1.90	1.44/-1.78	0.89/-1.92
CCDC	2330636	2330639	2377475

Supplementary Table 3. Key distances (Å) of experimental and DFT-optimized structures of **4**.

Bond length	Exp.	B3LYP		B3PW91[20]		PBE0		TPSS[21]	
		Cal.	RD	Cal.	RD	Cal.	RD	Cal.	RD
Pb1-Pb2	3.045	3.040	0.16%	3.036	0.30%	3.028	0.56%	3.066	0.69%
Sn1-Pb1	2.937	2.956	0.65%	2.943	0.20%	2.951	0.48%	2.977	1.36%
Sn2-Pb2	2.959	2.956	0.10%	2.943	0.54%	2.951	0.27%	2.977	0.61%
Sn1-N1	2.127	2.098	1.36%	2.093	1.60%	2.100	1.27%	2.111	0.75%
Sn1-N2	2.127	2.098	1.36%	2.094	1.55%	2.101	1.22%	2.114	0.61%
Sn1-N3	2.133	2.158	1.17%	2.162	1.36%	2.159	1.22%	2.18	2.20%
Sn2-N5	2.130	2.097	1.55%	2.093	1.74%	2.100	1.41%	2.111	0.89%
Sn2-N6	2.132	2.098	1.59%	2.094	1.78%	2.101	1.45%	2.114	0.84%
Sn2-N7	2.142	2.158	0.75%	2.161	0.89%	2.043	0.79%	2.044	1.77%
RD-Average		1.38%		1.11%		0.96%		1.08%	

$$RD = \frac{|BL_{Cal} - BL_{Exp}|}{BL_{Exp}} * 100\%$$

Where BL_{Cal} and BL_{Exp} represent the bond length measured by DFT calculation and experiment.

Supplementary Table 4. The relative free energy change (kcal/mol) of complex **4** in the singlet state (S0) and the triplet state (T1).

State	**4**-ΔG (kcal/mol)
S0	0.0
T1	23.0

Supplementary Table 5. Calculated UV-Vis absorption properties of complex **2**, including energy (eV), wavelength (nm), oscillator strength (f) and the transition with absolute contribution.

Energy/eV	Wavelength/nm	Oscillator strength/f	Transition with absolute contribution
2.68	462.8	0.0314	HOMO →LUMO (92.6%)
3.17	391.1	0.0925	HOMO →LUMO+2 (81.4%)<br>HOMO-1 →LUMO (6.4%)
3.28	378.1	0.0918	HOMO →LUMO+3 (88.0%)
3.36	369.2	0.0536	HOMO-1 →LUMO (88.0%)<br>HOMO →LUMO+2 (10.0%)

4. Supplementary References

1. Feng, G. et al. Transition-metal-bridged bimetallic clusters with multiple uranium-metal bonds. *Nat. Chem.* **11**, 248-253 (2019).

2. Sheldrick, G. M. Crystal structure refinement with SHELXL. *Acta Crystallogr. Sect. C*. **71**, 3-8 (2015).

3. Dolomanov, O. V., Bourhis, L. J., Gildea, R. J., Howard, J. A. K. & Puschmann, H. OLEX2: a complete structure solution, refinement and analysis program. *J. Appl. Crystallogr.* **42**, 339-341 (2009).

4. Frisch, M. J. et al. Gaussian 16, Revision A.03, Wallingford, CT, (2016).

5. Stephens, P. J., Devlin, F. J., Chabalowski, C. F. & Frisch, M. J. *Ab initio* calculation of vibrational absorption and circular dichroism spectra using density functional force fields. *J. Phys. Chem.* **98**, 11623-11627 (1994).

6. Petersson, G. A. et al. A complete basis set model chemistry. I. The total energies of closed-shell atoms and hydrides of the first-row elements. *J. Chem. Phys.* **89**, 2193-2221 (1988).

7. Rassolov, V. A., Pople, J. A., Ratner, M. A. & Windus, T. L. 6-31G* basis set for atoms K through Zn. *J. Chem. Phys.* **109**, 1223-1229 (1998).

8. Wadt, W. R. & Hay, P. J. Ab initio effective core potentials for molecular calculations. Potentials for main group elements Na to Bi. *J. Chem. Phys*. **82**, 284-298 (1985).

9. Ehlers, A. W. et al. A set of f-polarization functions for pseudo-potential basis sets of the transition metals Sc-Cu, Y-Ag and La-Au. *Chem. Phys. Lett.* **208**, 111-114 (1993).

10. Grimme, S., Ehrlich, S. & Goerigk, L. Effect of the damping function in dispersion corrected density functional theory. *J. Comput. Chem*. **32**, 1456-1465 (2011).

11. Hay, P. J., & Wadt, W. R. Ab initio effective core potentials for molecular calculations. Potentials for K to Au including the outermost core orbitals. *J. Chem. Phys.* **82**, 299–310 (1985).

12. Schwerdtfeger, P., Dolg, M., Schwarz, W. H. E., Bowmaker, G. A. & Boyd, P. D. W. Relativistic effects in gold chemistry. I. Diatomic gold compounds. *J. Chem. Phys*. **91**, 1762-1774 (1989).

13. Zhang, J. X., Sheong, F. K. & Lin, Z. Unravelling chemical interactions with principal interacting orbital analysis. *Chem.-Eur. J.* **24**, 9639-9650 (2018).

14. Zhang, J. X., Sheong, F. K. & Lin, Z. Principal interacting orbital: A chemically intuitive method for deciphering bonding interaction. *WIREs Comput. Mol. Sci*. **10**, e1469 (2020).

15. Glendening, E. D. et al. Theoretical Chemistry Institute, University of Wisconsin, Madison, WI, (2009).

16. Becke, A. D. Density-functional thermochemistry. III. The role of exact exchange. *J. Chem. Phys.* **98**, 5648-5652 (1993).

17. Cancès, E., Mennucci, B. & Tomasi, J. A new integral equation formalism for the polarizable continuum model: Theoretical background and applications to isotropic and anisotropic dielectrics. *J. Chem. Phys*. **107**, 3032-3041 (1997).

18. Lu, T. & Chen, F. Multiwfn: A multifunctional wavefunction analyser. *J. Comput. Chem*. **33**, 580-592 (2012).

19. Humphrey, W., Dalke, A. & Schulten, K. VMD: Visual molecular dynamics. *J. Mol. Graph. Model.* **14**, 33-38 (1996).

20. Salavati-Niasari, M., Mirsattari, S. N., Monajjemi, M. & Hamadanian, M. Density functional B3LYP and B3PW91 studies of the properties of four cyclic organodiboranes with tetramethylene fragments. *J Struct Chem* **51**, 437-443 (2010).

21. Tao, J., Perdew, J. P., Staroverov, V. N. & Scuseria, G. E. Climbing the density functional ladder: Nonempirical meta-generalized gradient approximation designed for molecules and solids. *Phys. Rev. Lett.* **91**, 146401 (2003).
